# Supplementary material for: F-actin disassembly by the oxidoreductase MICAL1 promotes mechano-dependent VWF-GPIbα interaction in platelets
Source: Nat Commun. 2025 Aug 10;16:7375. doi: 10.1038/s41467-025-62487-2 (PMC12335590; doi:10.1038/s41467-025-62487-2)
Supplement: Supplementary file 1 — Supplementary Information [file 41467_2025_62487_MOESM1_ESM.pdf]

F-actin disassembly by the oxidoreductase MICAL1 promotes mechano-dependent VWF-GPIbα interaction in platelets

Jean Solarz[1], Christelle Soukaseum[1], Stéphane Frémont[2], Sébastien Eymieux[3,4], Camilia Nabli[3], Christelle Repérant[1], Elisa Rossi[5], Jean-Claude Bordet[6], Cécile V. Denis[1,7], Pierre Mangin[8], Yacine Boulaftali[9], R Jeroen Pasterkamp[10], Hana Raslova[11], Dominique Baruch[12], Frédéric Adam[1,‡], Arnaud Echard[2,‡,*], Alexandre Kauskot[1,‡,*]

1 HITh, UMR_S1176, INSERM, Université Paris-Saclay, Le Kremlin-Bicêtre, France

2 Institut Pasteur, Université Paris Cité, CNRS UMR3691, Paris, France

3 Microscopy facility, US61 ASB, University of Tours, University Hospital Center of Tours, Inserm, Tours, France

4 INSERM U1259 MAVIVHe, University of Tours, Tours, France

5 Université Paris-Cité, INSERM, Optimisation thérapeutique en neuropharmacologie OTEN U1144, Paris, France

6 UR4609 Hémostase & Thrombose, Université Claude Bernard Lyon, Lyon, France

7 CHRU Nancy, Vandœuvre-lès-Nancy, France

8 INSERM U1255, Université de Strasbourg, EFS Grand-Est, Strasbourg, France

9 INSERM U1148, Université Paris Cité, Paris, France

10 Department of Translational Neuroscience, University Medical Center Utrecht, Brain Center, Utrecht University, Utrecht, The Netherlands

11 INSERM U1287, Institut Gustave Roussy, Université Paris Saclay, Villejuif, France

12 INSERM U1140, Université Paris Cité, Paris, France

‡ Contributed equally as senior authors

* Corresponding authors:

Alexandre KAUSKOT
INSERM U1176
Hôpital Le Kremlin-Bicêtre
Le Kremlin-Bicêtre
alexandre.kauskot@inserm.fr

Arnaud ECHARD
Institut Pasteur
CNRS 3691
Paris
arnaud.echard@pasteur.fr

SUPPLEMENTARY TABLES

Supplementary Table 1: Distribution of genotypes

There is non differences in genotype repartition. The groups were analyzed using a χ^2 test, p = 0.7292 (χ^2 =1.300, df=3)

Mical1^fl/fl^ **(*Mical1^+/+^*)**	***Mical1^fl/-^***	***Mical1^fl/fl; Pf4-Cre^*** **(*Mical1^-/-^*)**	***Mical1^fl/-; Pf4-Cre^***
128/541 (24%)	126/541 (23%)	137/541 (25%)	150/541 (28%)

Supplementary Table 2: Hematologic parameters in *Mical1$^{+/+}$* and *Mical1$^{-/-}$* mice separated by sex.

Mical1$^{-/-}$ mice show no differences in hematological parameters; platelet count and volume are normal. WBC: White Blood Cells; RBC: Red Blood Cells; MPV Mean Platelet Volume. (mean ± SD, N = number of mice, one-way ANOVA with Šídák post hoc test)

	Females			Males		
	Mical1$^{+/+}$	***Mical1$^{-/-}$***	***p***	***Mical1$^{+/+}$***	***Mical1$^{-/-}$***	***p***
WBC (x10^3/µL)	8.08 ± 1.91	8.36 ± 1.88	0.81	8.92 ± 1.13	8.84 ± 2.33	0.99
Lymphocytes (x10^3/µL)	6.81 ± 1.65	7.03 ± 1.58	0.84	7.38 ± 1.10	7.38 ± 2.05	0.99
Monocytes (x10^3/µL)	0.21 ± 0.07	0.22 ± 0.08	0.98	0.26 ± 0.06	0.26 ± 0.07	0.98
Granulocytes (x10^3/µL)	1.05 ± 0.27	1.11 ± 0.35	0.82	1.27 ± 0.49	1.21 ± 0.45	0.78
Eosinophils (x10^3/µL)	0.040 ± 0.01	0.039 ± 0.01	0.97	0.033 ± 0.02	0.044 ± 0.02	0.07
RBC (x10^6/µL)	9.51 ± 0.56	9.42 ± 0.51	0.76	9.58 ± 0.39	9.43 ± 0.30	0.42
Hemoglobin (g/dL)	14.21 ± 0.84	14.11 ± 0.58	0.82	14.10 ± 0.51	13.91 ± 0.39	0.48
Hematocrit (%)	46.5 ± 2.60	46.4 ± 2.38	0.99	46.8 ± 1.91	46.2 ± 2.15	0.57
N=	28	29		29	24	
Platelets (x10^3/µL)	856 ± 91.81	898 ± 145.40	0.23	989 ± 97.45	1009 ± 84.72	0.79
MPV (fL)	5.13 ± 0.17	5.08 ± 0.15	0.38	5.10 ± 0.13	5.09 ± 0.10	0.99
N=	34	33		29	24	

SUPPLEMENTARY FIGURES

Supplementary Figure 1

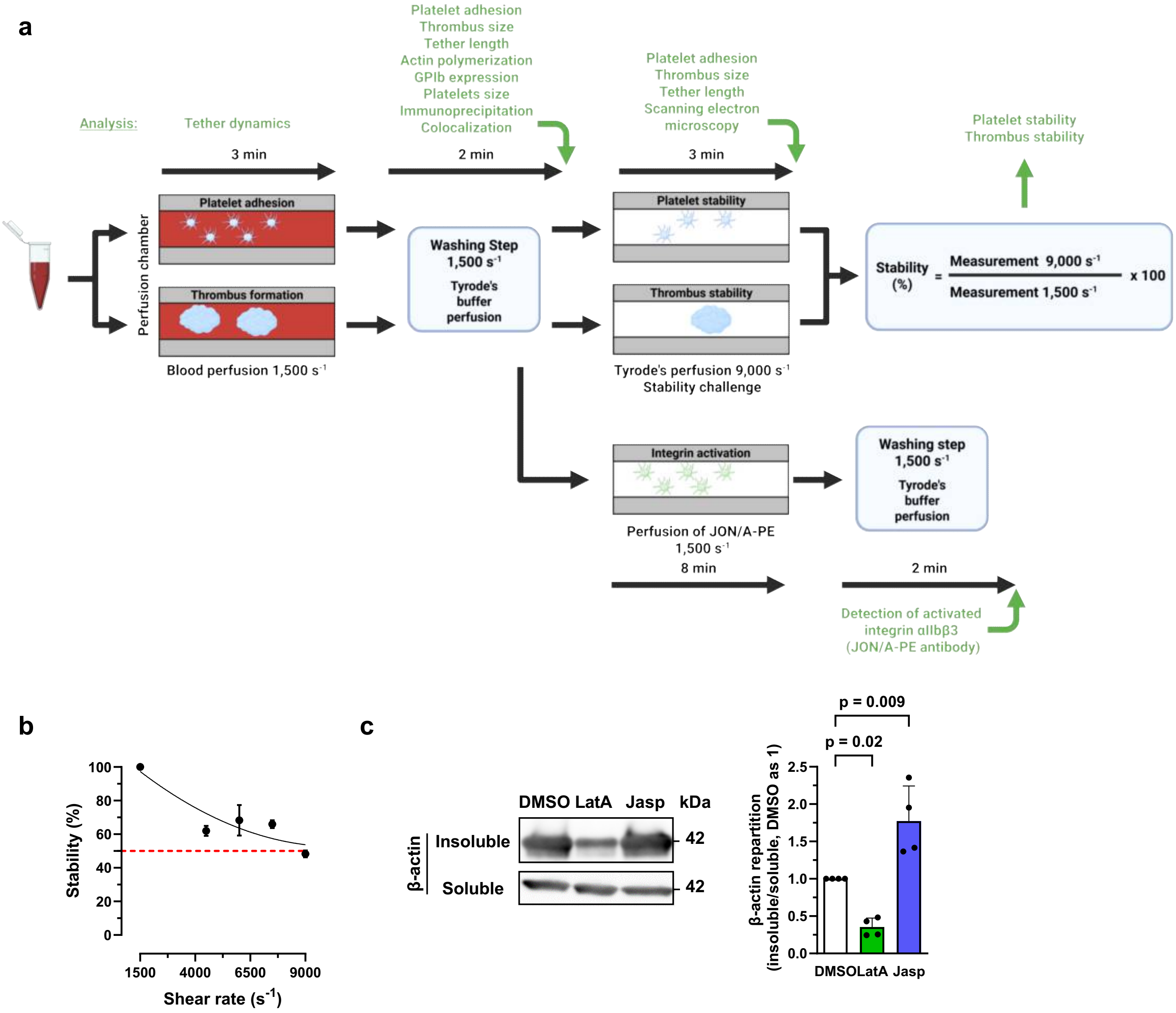

Supplementary Figure 1: Overview of flow experiments and measurement of the effect of Latrunculin-A and Jasplakinolide on actin polymerization

(a) Diagram depicting the flow assay protocol, which includes the blood perfusion step (shear rate of 1,500 s^{-1}), followed by a washing phase (shear rate of 1,500 s^{-1}) and a stability challenge (shear rate of 9,000 s^{-1}). The different analyses carried out in this study are indicated in green. *Created in BioRender. Solarz, J. (2025) https://BioRender.com/iyclvy4*

Stability and instability were calculated:

$$Platelet\ adhesion\ stability\ (\%) = \left[\frac{(Platelet\ adhesion\ (\%)\ at\ 9{,}000\ s^{-1})}{(Platelet\ adhesion\ (\%)\ at\ 1{,}500\ s^{-1})}\right] x\ 100$$

$$Thrombus\ stability\ (\%) = \left[\frac{(Thrombus\ size\ (\%)\ at\ 9{,}000\ s^{-1})}{(Thrombus\ size\ (\%)\ at\ 1{,}500\ s^{-1})}\right] x\ 100$$

$$Thrombus\ instability\ (\%) = (1 - Thrombus\ stability)$$

(b) Thrombus stability was evaluated at various shear rates on collagen matrix. Dashed red line indicates 50% of stability.
(c) Mouse platelets were treated with either Latrunculin-A (LatA; 500 nM), Jasplakinolide (Jasp; 1 µM), or DMSO as a control, lysed and β-actin was analyzed in both the Triton-X100 insoluble and soluble fractions to assess the effects of the drugs on actin solubility, reflecting polymerization or depolymerization status. Of note, F-actin is incorporated into the Triton-X100 insoluble fraction. (mean ± SD, N = 4 independent experiments from 4 different mice, one-way ANOVA with Tukey post hoc test, $F = 12.33$, $df = 11$). Source data are provided as a Source Data file

Supplementary figure 2

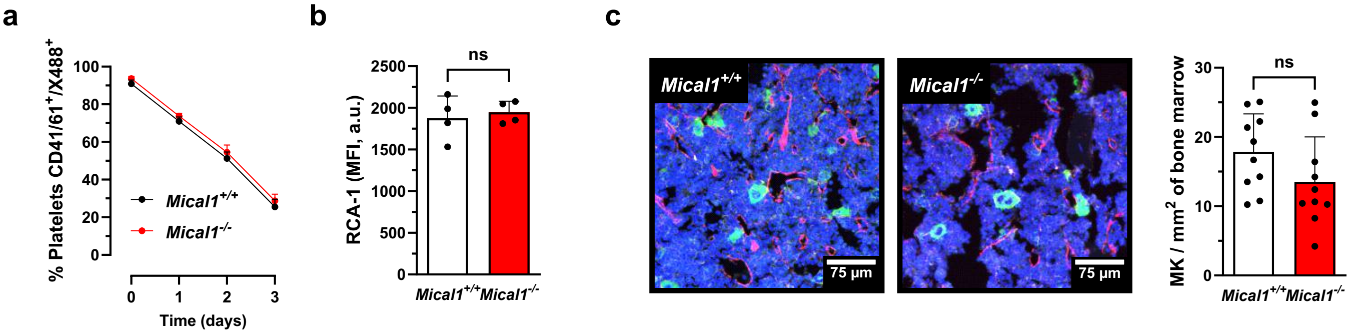

Supplementary Figure 2: MICAL1 deficiency does not affect platelet lifespan or the number of megakaryocytes in the bone marrow

(a) Platelet clearance in *Mical1*$^{+/+}$ and *Mical1*$^{-/-}$ mice was measured with labelled fluorescent anti-GPIX antibody. The percentage of labelled platelets (X488^{+}) was monitored by flow cytometry in the CD41/61^{+} population (mean ± SD, N: *Mical1*$^{+/+}$ = 4 independent experiments from 4 different mice, *Mical1*$^{-/-}$ = 3, two-way ANOVA with Šídák post hoc test, F(3, 20) = 0.01732).
(b) Platelet b-galactose exposure of *Mical1*$^{+/+}$ and *Mical1*$^{-/-}$ platelets was evaluated with RCA-1 lectin by flow cytometry (mean fluorescence intensity (MFI) ± SD, N = 4 independent experiments from 4 different mice, two-tailed unpaired Student's t-test, t = 0.4755, df = 6).
(c) Images of bone marrow megakaryocytes (MK) in *Mical1*$^{+/+}$ and *Mical1*$^{-/-}$ mice detected by immunofluorescence using CD41 antibody (mean of megakaryocytes per mm² ± SD, N = 10 fields corresponding to the whole femur of 3 different mice, two-tailed unpaired Student's t-test, t = 1.956, df = 18). Left panel: representative images of bone marrow immunofluorescence, blue: DNA, green: CD41, red: Laminin.
ns: not significant.

Supplementary figure 3

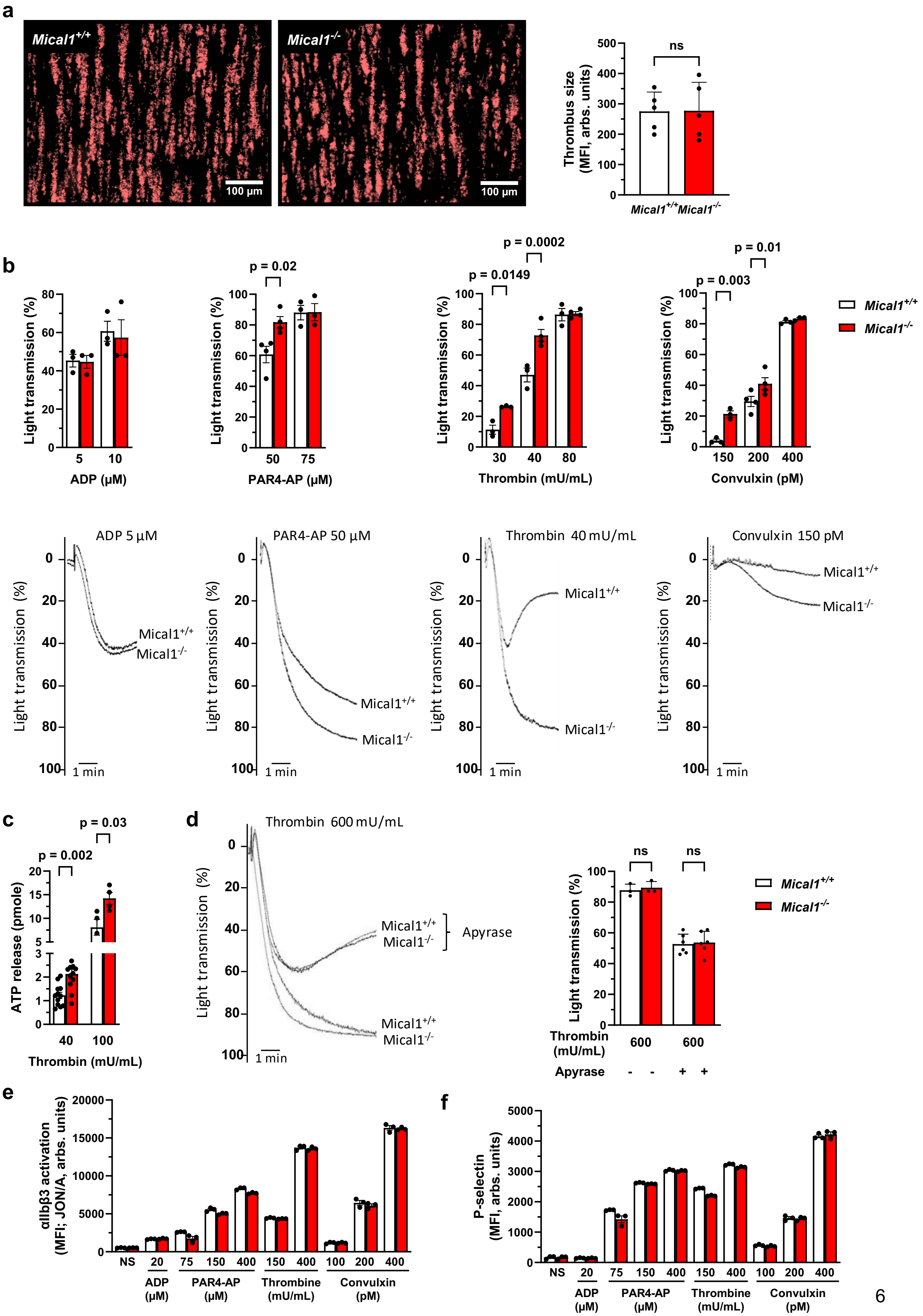

Supplementary Figure 3: MICAL1 deficiency increases platelet aggregation in a secretion-dependent manner

(a) Rhodamine 6G stained *Mical1*$^{+/+}$ or *Mical1*$^{-/-}$ platelets in whole blood treated with 5 U/mL of apyrase were perfused on type I collagen matrix. Thrombus size at a shear rate of 1,500 s^{-1} (mean fluorescence intensity (MFI) ± SD, N = 5; two-tailed paired Student's t-test, t = 0.04228, df = 8). Left panel: representative images.
(b) Aggregation of washed *Mical1*$^{+/+}$ and *Mical1*$^{-/-}$ platelets activated with different agonists (ADP: mean ± SD, N = 3, multiple unpaired Student's t-test, t: 5 µM = 0.08071, 10 µM = 0.4036, df = 8) (PAR4-AP: mean ± SD, N: 50 µM = 4; 75 µM= 3, multiple unpaired Student's t-test, t: 50 µM = 3.256, 75 µM = 0.04475, df = 10) (Thrombin: mean ± SD, N: 30 mU/mL = 3; 40 and 80 mU/mL = 4, multiple unpaired Student's t-test, t: 30 mU/mL = 3.122, 40 mU/mL = 5.730, 80 mU/mL = 0.1483, df = 14) (Convulxin: mean ± SD, N: 150 pM = 3, 200 pM = 4; * $p < 0.01$, 400 pM = 3, multiple unpaired Student's t-test, t: 150 pM = 4.216, 200 pM = 3.230, 400 pM = 0.4864, df = 14). Bottom panels: representative aggregation traces.
(c) Measure of ATP release during thrombin aggregation (40 and 100 mU/mL) (mean ± SD, N: 40 mU/mL = 12, 100 mU/mL = 4, multiple unpaired Student's t-test, 40 mU/mL: t = 3.875, df = 19.38,100 mU/mL: t = 2.923, df= 5.570).
(d) Aggregation of washed *Mical1*$^{+/+}$ and *Mical1*$^{-/-}$ platelets activated with thrombin 600 mU/mL after incubation with 2 U/mL of apyrase or not (mean ± SD, in absence of apyrase N = 3, in presence of apyrase N = 6, multiple unpaired Student t-test, t: without apyrase (-) = 0.3269, with apyrase (+) = 0.2774, df = 14). Left panel: representative aggregation traces.
(e-f) Washed *Mical1*$^{+/+}$ and *Mical1*$^{-/-}$ platelets were activated with different agonists as indicated in the panel.
(e) $\alpha IIb\beta_3$ activation was monitored by flow cytometry with JON/A antibody (mean ± SD, N = 3, multiple paired Student t-test, t: NS = 0.1278, ADP 20 µM = 1.554, PAR4-AP 75 µM = 2.888, PAR4-AP 150 µM = 3.550, PAR4-AP 400 µM = 10.84, thrombin 150 mU/mL = 2.123, thrombin 400 mU/mL = 0.6290, convulxin 100 pM = 0.05612, convulxin 200 pM = 0.7085, convulxin 400 pM = 0.4367, df = 2). (f) Platelet secretion of α-granules was monitored with P-selectin exposure using specific antibody (mean ± SD, N = 3, multiple paired Student t-test, t: NS = 1.055, ADP 20 µM = 0.5371, PAR4-AP 75 µM = 2.584, PAR4-AP 150 µM = 6.9, PAR4-AP 400 µM = 1.024, thrombin 150 mU/mL = 8.644, thrombin 400 mU/mL = 4.799, convulxin 100 pM = 1.128, convulxin 200 pM = 0.2424, convulxin 400 pM = 0.5482, df = 2). NS: non-stimulated platelets.
ns: not significant. MFI: mean fluorescence intensity. N = number of independent experiments from different mice.

Supplementary figure 4

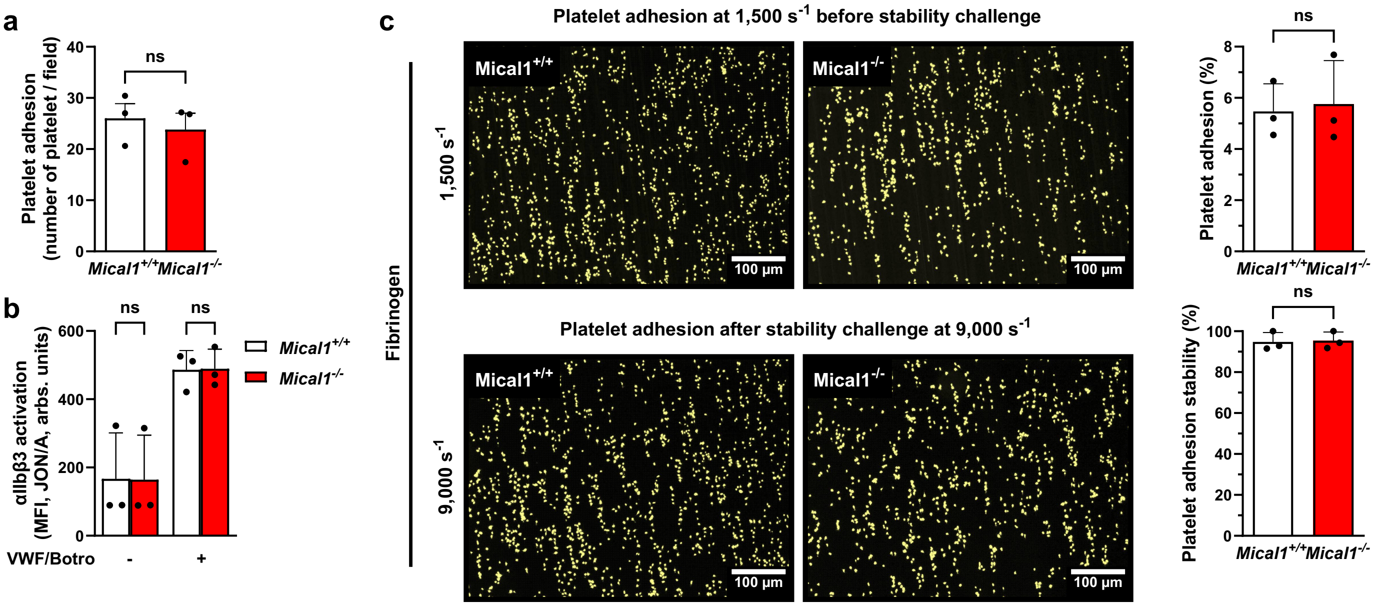

Supplementary Figure 4: MICAL1 deficiency does not affect shear-dependent platelet adhesion over fibrinogen

(a) Platelet adhesion of *Mical1*$^{+/+}$ and *Mical1*$^{-/-}$ washed platelets deposited on recombinant mouse VWF matrix (static) with botrocetin (5 µg/mL). Adherent platelets were labeled with phalloidin and observed by immunofluorescence microscopy (mean ± SD, N = 3, two-tailed unpaired Student's t-test, t = 0.5119, df = 4).

(b) αIIbβ$_3$ activation by VWF (10 µg/mL) and botrocetin (5 µg/mL) was monitored by flow cytometry with JON/A antibody (mean fluorescence intensity (MFI) ± SD, N = 3, two-way ANOVA, F(1, 8) = 0.002080) in *Mical1*$^{+/+}$ and *Mical1*$^{-/-}$ platelets.

(c) Blood from *Mical1*$^{+/+}$ or *Mical1*$^{-/-}$ mice was stained with rhodamine 6G and perfused over a fibrinogen matrix. Platelet adhesion (top panels) (mean ± SD, N = 3, two-tailed unpaired Student's t-test, t = 0.2451, df = 4) and platelet stability (bottom panels) were measured (mean ± SD, N = 3, two-tailed unpaired Student's t-test, t = 0.1679, df = 4). Left panels: representative images showing platelet adhesion and stability. Scale bars: 100 µm.

ns: not significant. N = number of independent experiments from different mice.

Supplementary figure 5

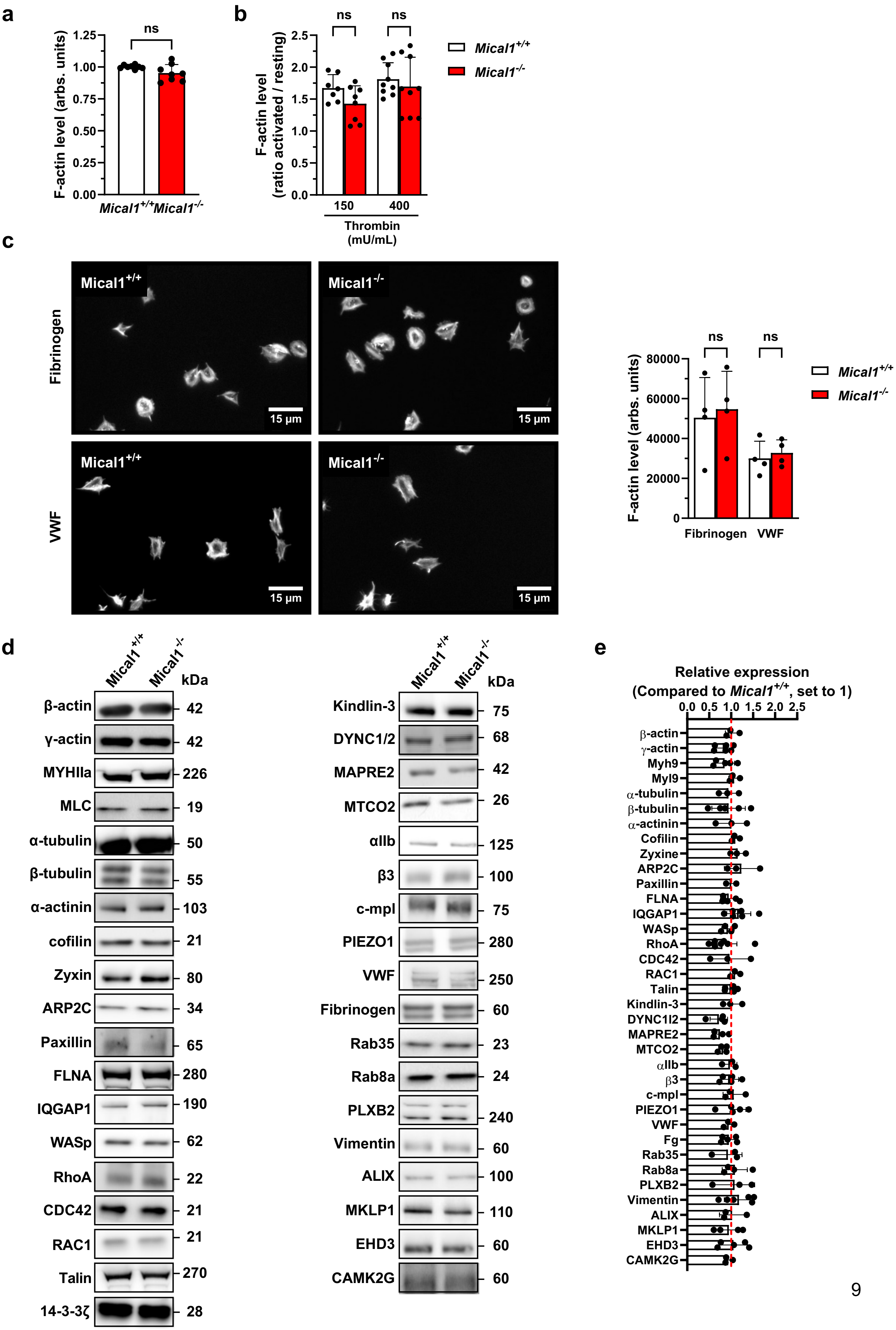

Supplementary Figure 5: MICAL1 deficiency does not affect F-actin polymerization in static conditions and does not impact the expression of main actin regulators and other proteins related to platelets or MICAL1

(a-b) F-actin levels from *Mical1*$^{+/+}$ and *Mical1*$^{-/-}$ platelets were measured by flow cytometry in (a) resting platelets (mean fluorescence intensity (MFI) ± SD, N = 8, two-tailed unpaired t-test, t = 1.992, df = 14) and in (b) thrombin (150 and 400 mU/mL) activated platelets (mean fluorescence intensity (MFI) ± SD, N: 150 mU/mL: *Mical1*$^{+/+}$ = 7, *Mical1*$^{-/-}$ = 8, 400 mU/mL = 9, two-way ANOVA with Šídák post hoc test, F(1, 29) = 0.3333), expressed as ratio of activated to resting platelets.
(c) F-actin levels in *Mical1*$^{+/+}$ and *Mical1*$^{-/-}$ platelets measured by fluorescence microscopy on either fibrinogen or recombinant mouse VWF matrix (mean fluorescence intensity (MFI) ± SD, N: fibrinogen = 3, VWF = 4, two-way ANOVA with Šídák post hoc test, F(1, 12) = 0.01047). Left panels: representative images of fluorescent microscopy showing phalloidin-stained platelets, scale bars: 15 µm.
(d) Representative images of western blots of lysates from *Mical1*$^{+/+}$ and *Mical1*$^{-/-}$ platelets.
(e) Relative expression of proteins in *Mical1*$^{-/-}$ compared to *Mical1*$^{+/+}$ set to 1 (red dashed line). (N: β-actin = 3, γ-actin = 6, Myh9 = 5, Myl9 = 3, α-tubulin = 3, β-tubulin = 5, α-actinin = 3, Cofilin = 3, Zyxine = 3, ARP2C = 3, Paxillin = 2, FLNA = 6, IQGAP1 = 6, WASp = 4, RhoA = 8, CDC42 = 3, RAC1 = 3, Talin = 5, Kindlin-3 = 3, DYNC1/2 = 4, MAPRE2 = 4, MTCO2 = 4, αIIb = 3, β3 = 5, MPL = 3, PIEZO1 = 5, Fg= 5, Rab35 = 3, Rab8a = 4, PLXB2 = 3, Vimentin = 6, ALIX = 3, MKLP1 = 4, EHD3 = 5, CAMK2G = 3, with 14-3-3z as loading control).
ns: not significant. N = number of independent experiments from different mice.
Source data are provided as a Source Data file

Supplementary figure 6

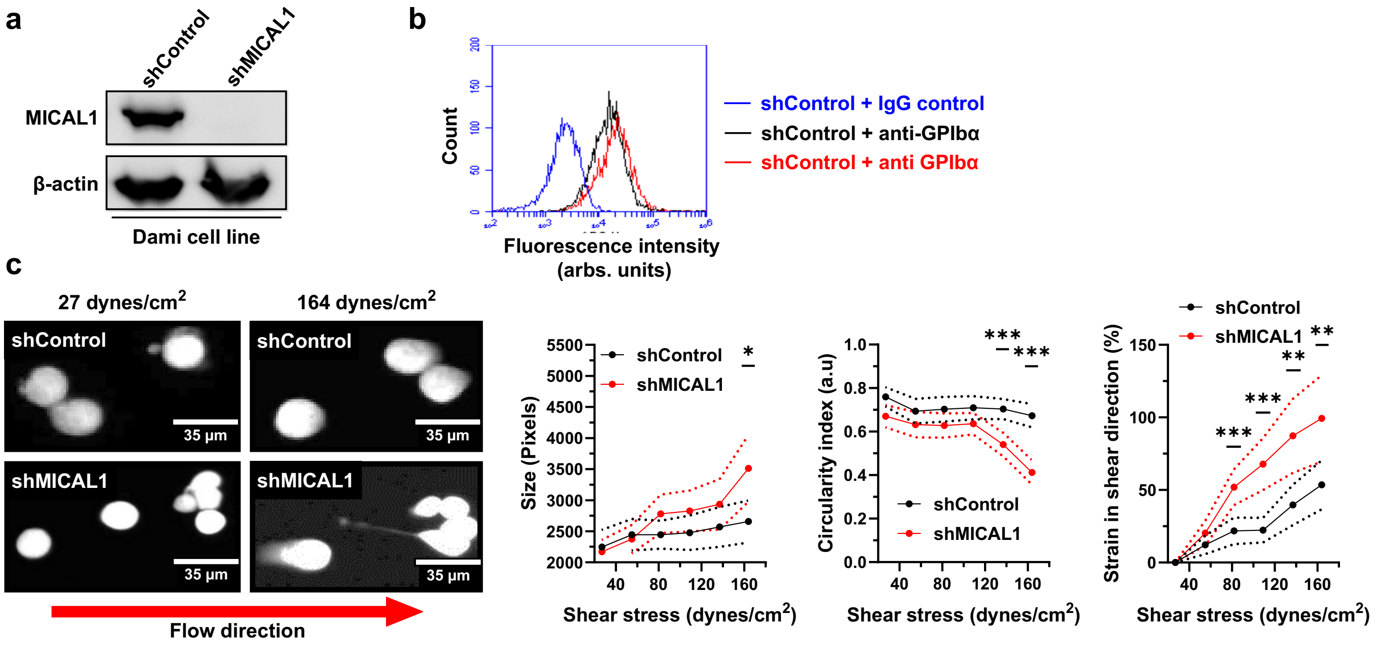

Supplementary Figure 6: MICAL1 depletion in DAMI cells does not alter GPIbα expression but showed a marked increase in size, shear strain and loss of circularity upon shear

(a) Lysates from ShControl and ShMICAL1 DAMI cells were blotted for MICAL1 and b-actin as loading control. Source data are provided as a Source Data file
(b) GPIbα surface expression measured by flow cytometry in ShControl and ShMICAL1 DAMI cells.
(c) GFP-DAMI cells, shControl, and shMICAL1 were perfused on human VWF matrix. After adhesion, cells were exposed to a shear gradient ranging from 27 to 164 dynes/cm^2. Cell deformation (Size, Circularity index, and strain) was evaluated in multiple cells from 3 independent experiments. Left panels: representative images showing cell deformation. Scale bars: 35 µm. N = number of cells.
Curves of cell size (mean ± 95% CI, N: 27-55 dynes/cm^2: shControl = 41 and shMICAL1 = 45; 82 dynes/cm^2: shControl = 41 and shMICAL1 = 43; 109 dynes/cm^2: shControl = 36 and shMICAL1 = 41; 137dynes/cm^2: shControl = 32 and shMICAL1 = 41; 164 dynes/cm^2: shControl = 31 and shMICAL1 = 34, mixed-effects analysis, F(5, 375) = 6.553).
Curves of circularity index (mean ± 95% CI, N: 27-55 dynes/cm^2: shControl = 41 and shMICAL1 = 45; 82 dynes/cm^2: shControl = 41 and shMICAL1 = 43; 109 dynes/cm^2: shControl = 36 and shMICAL1 = 41; 137 dynes/cm^2: shControl = 32 and shMICAL1 = 41, *** $p < 0.001$; 164 dynes/cm^2: shControl = 31 and shMICAL1 = 34, mixed-effects analysis, F(5, 375) = 4.134).
Curves of shear strain (mean ± 95% CI, N: 27-55 dynes/cm^2: shControl = 43 and shMICAL1 = 49; 82 dynes/cm^2: shControl = 42 and shMICAL1 = 48, *** $p < 0.001$; 109 dynes/cm^2: shControl = 37 and shMICAL1 = 47, *** $p < 0.001$; 137dynes/cm^2: shControl = 33 and shMICAL1 = 43, ** $p = 0.001$; 164 dynes/cm^2: shControl = 32 and shMICAL1 = 35, mixed-effects analysis, F(5, 399) = 8.609).

Supplementary Figure 7

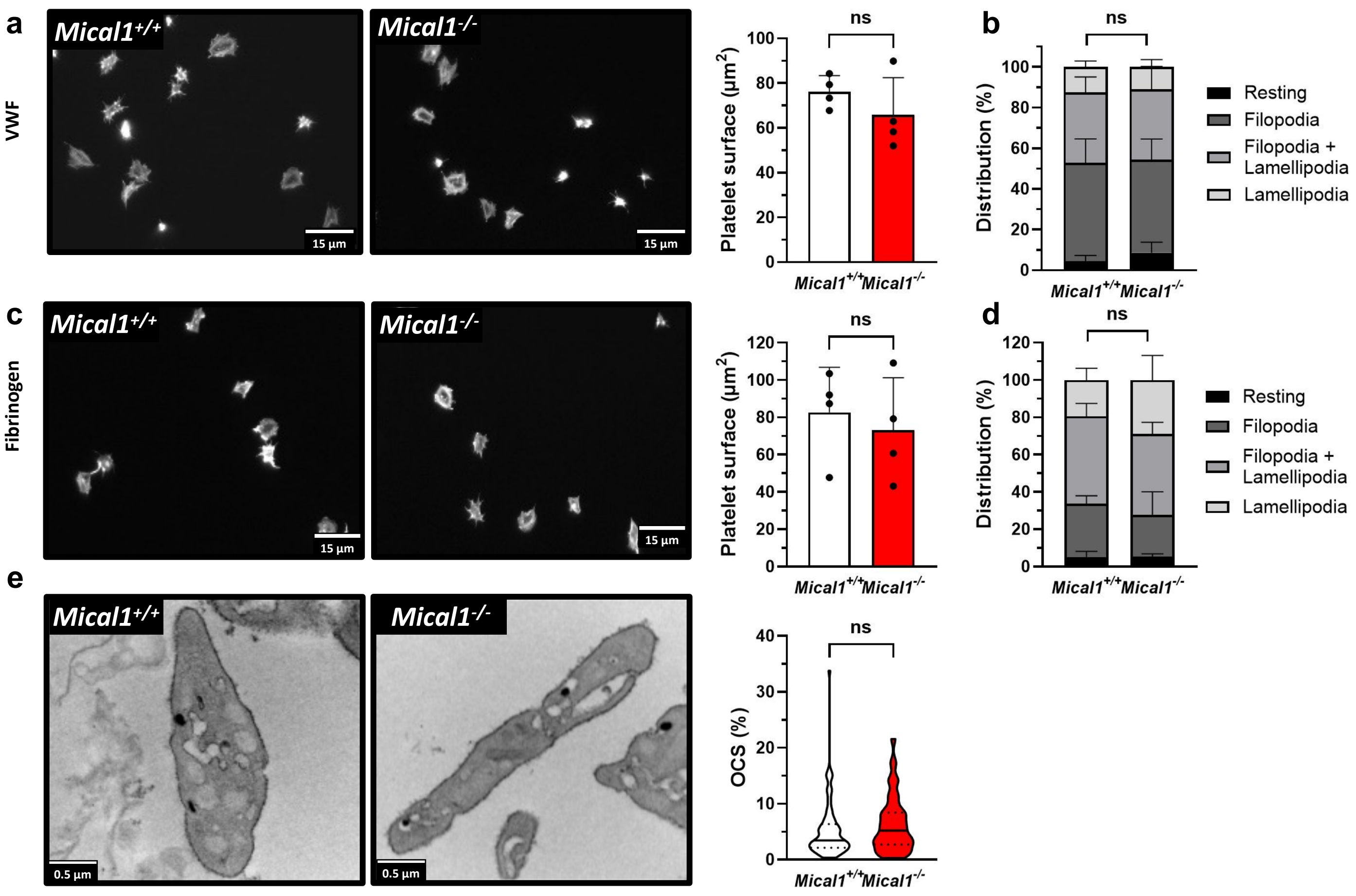

Supplementary Figure 7: In static conditions, MICAL1 is not involved in the F-actin dynamics and does not alter OCS formation

(a-d) Images of fluorescent microscopy of *Mical1*$^{+/+}$ and *Mical1*$^{-/-}$ platelets labeled with fluorescent phalloidin on recombinant mouse VWF or fibrinogen matrix. (a) Platelet area (mean ± SD, N = 4 independent experiments from 4 different mice, two-tailed unpaired Student's t-test, t = 1.151, df = 6) and (b) morphological repartition of adherent platelets to recombinant mouse VWF matrix (mean ± SD, N = 4, two-way ANOVA with Šídák post hoc test, F(3, 24) = 0.2755). Left panel: representative images of platelet size and their morphology on recombinant mouse VWF matrix. Scale bars: 15 µm. (c) Platelet area (mean ± SD, N = 4 independent experiments from 4 different mice, two-tailed unpaired Student's t-test, t = 0.5163, df =6) and (d) platelet morphology repartition on fibrinogen matrix (mean ± SD, N = 3 independent experiments from 4 different mice, two-way ANOVA with Šídák post hoc test, F(3, 16) = 1.178). Left panels: representative images of platelet size and morphology on fibrinogen matrix. Scale bars: 15 µm. (e) Open canalicular system (OCS) by transmission electron microscopy with ruthenium staining. Violin plots with the median represented by a central line and the interquartile range (25th-75th percentiles) indicated by the upper and lower lines. (N: *Mical1*$^{+/+}$ = 84, *Mical1*$^{-/-}$ = 83 platelets, two-way unpaired Student's t-test, t = 1.427, df =165). Left panels: representative images of platelets OCS. Scale bars: 0.5 µm.
ns: not significant.

Supplementary Figure 8

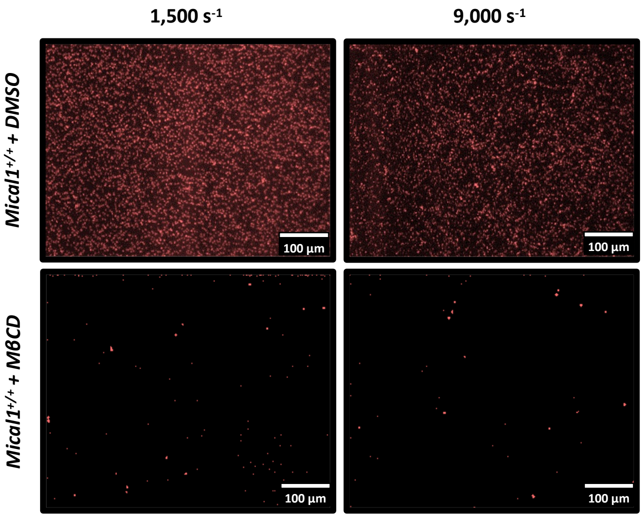

Supplementary Figure 8: Cholesterol depletion inhibits platelet adhesion to VWF

Platelet rich plasma (PRP) from wild type mice (Mical1$^{+/+}$) was isolated from anticoagulated blood, incubated for 30 min at 37°C with methyl-β-cyclodextrin (MβCD, 30 mM) to deplete cholesterol and to disrupt lipid rafts or with DMSO, as control. Platelets were stained with rhodamine 6G prior to reconstitution to normal hematocrit values with red blood cells. Reconstituted blood was perfused on recombinant mouse VWF. Representative image of platelet adhesion after lipid raft depletion with MβCD. Scale bars: 100 µm. N = 1 experiment.

Supplementary Figure 9

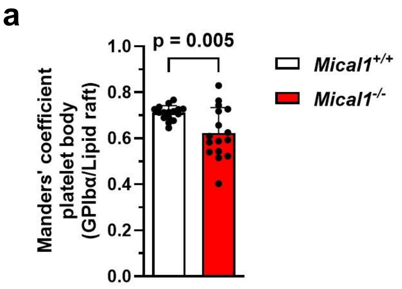

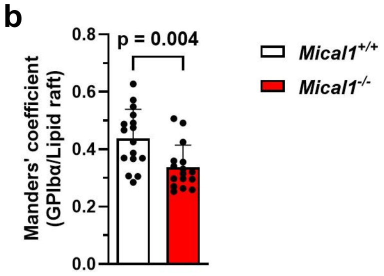

Supplementary Figure 9: MICAL1 modulates GPIbα and lipid rafts colocalization in the platelet body and tether

Colocalization of lipid rafts (CTxB) and GPIbα in *Mical1*$^{+/+}$ and *Mical1*$^{-/-}$ platelets after flow assays at 1,500s^{-1} from 3 independent experiments corresponding to different mice. (a) Manders' overlap coefficient for *Mical1*$^{+/+}$ vs *Mical1*$^{-/-}$ platelets within the platelet body (mean ± SD, N = 16 fields from 3 independent experiments for both genotypes, two-tailed unpaired Student's t-test, t = 3.044, df = 30) (b) Manders' overlap coefficient for *Mical1*$^{+/+}$ vs *Mical1*$^{-/-}$ platelets in the platelet tether (mean ± SD, N = 16 fields from 3 independent experiments for both genotypes, two-tailed unpaired Student's t-test, t = 3.164, df = 30).

Supplementary Figure 10

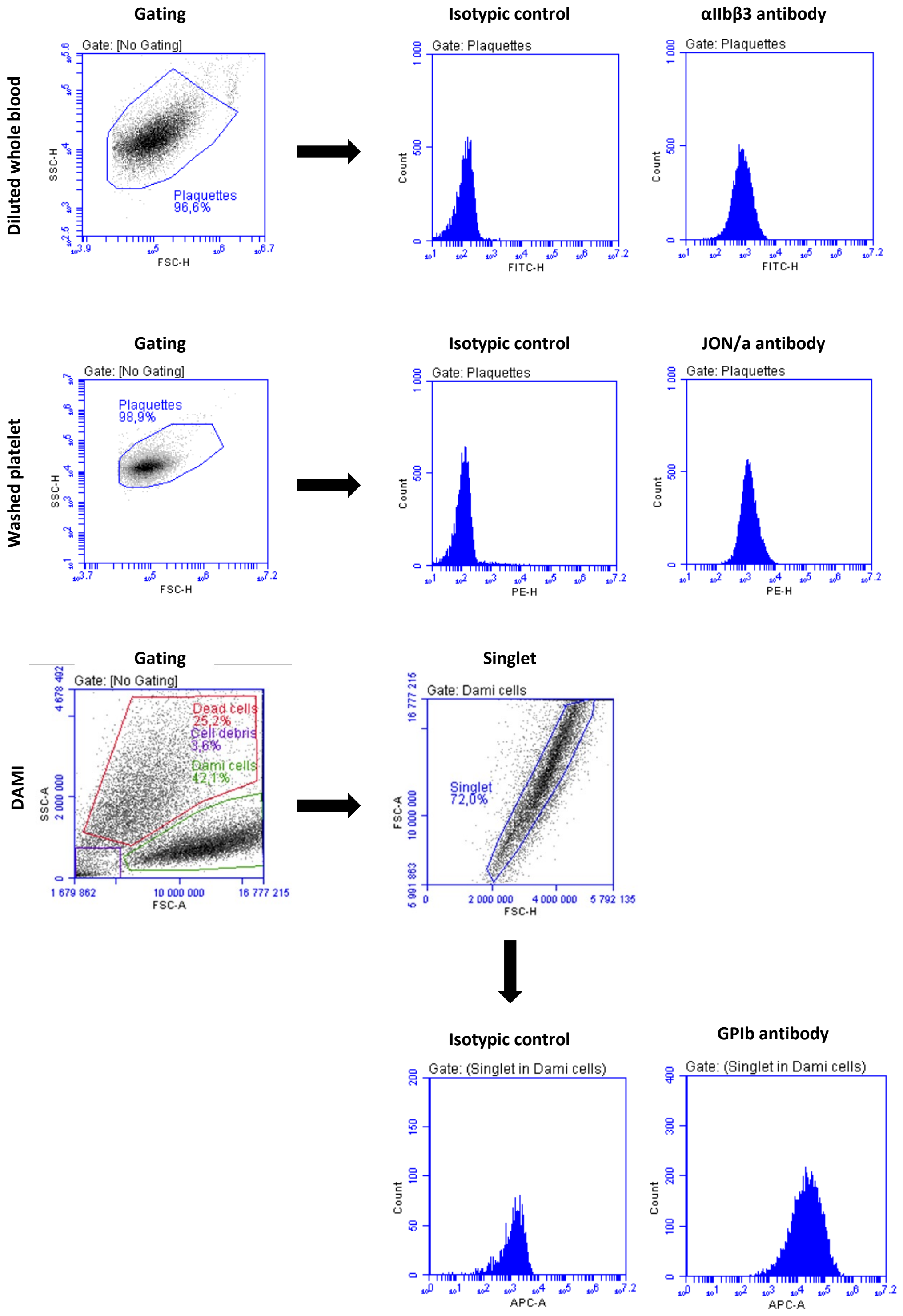

Supplementary Figure 10: Gating strategy used by flow cytometry to analyze platelets and DAMI cell line.
Platelets in diluted whole blood (Fig. 3, Supplementary Fig. 2), washed platelets (Fig. 1, 7, Supplementary Fig. 3, 5) and DAMI cell line (Supplementary Fig. 6)
