# Supplementary material for: F-actin disassembly by the oxidoreductase MICAL1 promotes mechano-dependent VWF-GPIbα interaction in platelets
Source: Nat Commun. 2025 Aug 10;16:7375. doi: 10.1038/s41467-025-62487-2 (PMC12335590; doi:10.1038/s41467-025-62487-2)
Supplement: Supplementary file 2 — Description of Additional Supplementary File [file 41467_2025_62487_MOESM2_ESM.pdf]

Additional Description of Supplementary Files

Supplementary Video File 1: Rolling experiment of mouse platelets treated with DMSO.

Supplementary Video File 2: Rolling experiment of mouse platelets treated with LatA.

Supplementary Video File 3: Rolling experiment of mouse platelets treated with Jasp

Supplementary Video File 4: Tether dynamics of Mical1+/+ platelets.

Supplementary Video File 5: Tether dynamics of Mical1-/- platelets.

Supplementary Video File 6: Rolling experiment of Mical1+/+ platelets.

Supplementary Video File 7: Rolling experiment of Mical1-/- platelets.

Supplementary Video File 8: Rolling experiment of Mical1+/+ platelets treated with DMSO.

Supplementary Video File 9: Rolling experiment of Mical1+/+ platelets treated with LatA.

Supplementary Video File 10: Rolling experiment of Mical1-/- platelets treated with DMSO.

Supplementary Video File 11: Rolling experiment of Mical1-/- platelets treated with a low dose of LatA (rescue experiment).

Supplementary Data 1: Reagent list

Supplementary File 2: Proteins list from Biogrid and Gene Ontology “Actin filament depolymerization” (GO: 0030042)
