# Supplementary material for: F-actin disassembly by the oxidoreductase MICAL1 promotes mechano-dependent VWF-GPIbα interaction in platelets
Source: Nat Commun. 2025 Aug 10;16:7375. doi: 10.1038/s41467-025-62487-2 (PMC12335590; doi:10.1038/s41467-025-62487-2)
Supplement: Supplementary file 16 — Reporting summary [file 41467_2025_62487_MOESM16_ESM.pdf]

nature portfolio

Corresponding author(s): Alexandre Kauskot, Arnaud Echard

Last updated by author(s): Jun 16, 2025

Reporting Summary

Nature Portfolio wishes to improve the reproducibility of the work that we publish. This form provides structure for consistency and transparency in reporting. For further information on Nature Portfolio policies, see our Editorial Policies and the Editorial Policy Checklist.

Statistics

For all statistical analyses, confirm that the following items are present in the figure legend, table legend, main text, or Methods section.

n/a	Confirmed	
☐	☒	The exact sample size (*n*) for each experimental group/condition, given as a discrete number and unit of measurement
☐	☒	A statement on whether measurements were taken from distinct samples or whether the same sample was measured repeatedly
☐	☒	The statistical test(s) used AND whether they are one- or two-sided<br>*Only common tests should be described solely by name; describe more complex techniques in the Methods section.*
☐	☒	A description of all covariates tested
☐	☒	A description of any assumptions or corrections, such as tests of normality and adjustment for multiple comparisons
☐	☒	A full description of the statistical parameters including central tendency (e.g. means) or other basic estimates (e.g. regression coefficient) AND variation (e.g. standard deviation) or associated estimates of uncertainty (e.g. confidence intervals)
☐	☒	For null hypothesis testing, the test statistic (e.g. *F*, *t*, *r*) with confidence intervals, effect sizes, degrees of freedom and *P* value noted<br>*Give P values as exact values whenever suitable.*
☒	☐	For Bayesian analysis, information on the choice of priors and Markov chain Monte Carlo settings
☒	☐	For hierarchical and complex designs, identification of the appropriate level for tests and full reporting of outcomes
☐	☒	Estimates of effect sizes (e.g. Cohen's *d*, Pearson's *r*), indicating how they were calculated

Our web collection on statistics for biologists contains articles on many of the points above.

Software and code

Policy information about availability of computer code

Data collection	Data were collected using: Excel (mac version 2018), automated cell counter (Scil Vet ABC Plus, Horiba Medical), thrombosis model: blood flow with a perivascular flow probe (TS420 Perivascular Flow Module, Transonic System Inc, Ithaca, NY), microscopy: inverted epifluorescence microscope (Nikon Eclipse TE2000-U), epifluorescence microscopy (Eclipse Nikon 600), an inverted Nikon Eclipse Ti-E microscope equipped with a CSU-X1 spinning disk confocal scanning unit (Yokogawa) coupled to a Prime 95S scientific complementary metal-oxide semiconductor (sCMOS) camera (Teledyne Photometrics) using a X 100 1.4 NA CFI Plan APO VC objective lens, JEOL JEM1400 transmission electron microscope with a Gatan Orius 600 camera, Zeiss Ultra plus FEG-SEM scanning electron microscope (Oberkochen), confocal microscopy (Confocal Laser Scanning Microscope, CLSM, Leica TCS SP5, Leica Biosystems France) flow cytometry: Accuri C6 cytometer (BD Biosciences), westernblot: chemiluminescent signal were captured using G:BOX Chemi XT16 Image Systems or with the Amersham Typhoon, Chronolog aggregometer model 700 (Chrono-log Corporation), luminometer (Fluoroskan Ascent FL; Thermo LabSystems), automated capillary-based immunoassay platform Wes (ProteinSimple)
Data analysis	MetaMorph 7.0r1 software (Molecular Devices), GraphPad Prism 10.5.0 (Dotmatics, USA), ImageJ 19.13.33, Gene Tools version 4.0.0.0 (Syngene), ImageQuant TL image analysis software (GE Healthcare Bio-sciences AB), LabChart Reader 8 software (AD instrument), AGGRO/LINK®8, C6 plus analysis software (BD Biosciences)

For manuscripts utilizing custom algorithms or software that are central to the research but not yet described in published literature, software must be made available to editors and reviewers. We strongly encourage code deposition in a community repository (e.g. GitHub). See the Nature Portfolio guidelines for submitting code & software for further information.

Data

Policy information about availability of data

All manuscripts must include a data availability statement. This statement should provide the following information, where applicable:

- Accession codes, unique identifiers, or web links for publicly available datasets
- A description of any restrictions on data availability
- For clinical datasets or third party data, please ensure that the statement adheres to our policy

The authors declare that the main data supporting the findings of this study are available within the article and its Supplementary Information files.

Research involving human participants, their data, or biological material

Policy information about studies with human participants or human data. See also policy information about sex, gender (identity/presentation), and sexual orientation and race, ethnicity and racism.

Reporting on sex and gender	*Use the terms sex (biological attribute) and gender (shaped by social and cultural circumstances) carefully in order to avoid confusing both terms. Indicate if findings apply to only one sex or gender; describe whether sex and gender were considered in study design; whether sex and/or gender was determined based on self-reporting or assigned and methods used. Provide in the source data disaggregated sex and gender data, where this information has been collected, and if consent has been obtained for sharing of individual-level data; provide overall numbers in this Reporting Summary. Please state if this information has not been collected. Report sex- and gender-based analyses where performed, justify reasons for lack of sex- and gender-based analysis.*
Reporting on race, ethnicity, or other socially relevant groupings	*Please specify the socially constructed or socially relevant categorization variable(s) used in your manuscript and explain why they were used. Please note that such variables should not be used as proxies for other socially constructed/relevant variables (for example, race or ethnicity should not be used as a proxy for socioeconomic status). Provide clear definitions of the relevant terms used, how they were provided (by the participants/respondents, the researchers, or third parties), and the method(s) used to classify people into the different categories (e.g. self-report, census or administrative data, social media data, etc.) Please provide details about how you controlled for confounding variables in your analyses.*
Population characteristics	*Describe the covariate-relevant population characteristics of the human research participants (e.g. age, genotypic information, past and current diagnosis and treatment categories). If you filled out the behavioural & social sciences study design questions and have nothing to add here, write "See above."*
Recruitment	*Describe how participants were recruited. Outline any potential self-selection bias or other biases that may be present and how these are likely to impact results.*
Ethics oversight	*Identify the organization(s) that approved the study protocol.*

Note that full information on the approval of the study protocol must also be provided in the manuscript.

Field-specific reporting

Please select the one below that is the best fit for your research. If you are not sure, read the appropriate sections before making your selection.

☒ Life sciences ☐ Behavioural & social sciences ☐ Ecological, evolutionary & environmental sciences

For a reference copy of the document with all sections, see nature.com/documents/nr-reporting-summary-flat.pdf

Life sciences study design

All studies must disclose on these points even when the disclosure is negative.

Sample size	A power analysis was performed using G*Power software was performed to ensure that the sample size was sufficient to detect statistically significant differences with a power of 80% and a significance level of 0.05. All mouse procedures, including the statistics and the number of mice necessary for each procedure have been validated and approved by the local ethical committee CEEA26 and the French government under the number APAFIS#25086-2020032312267714.
Data exclusions	No exclusion
Replication	Western blots were performed 3-5 times with platelets from at least 2 mice per genotype with similar results. Flow cytometry experiments were repeatd 3-5 times with platelet from at least 2 mice per genotype with similar results. In vivo experiments were repeated 3-7 times with similar results; all in vitro assays were repeated at least 3 times to demonstrate statistical significance or absence of it. Number of replicated of each experiment is indicated in the corresponding figure legend.
Randomization	No randomization

Blinding	For all experiments automated quantitative methods were used to avoid researchers bias. In vivo thrombosis models and tail clip were performed in blind (genotype of mice) by the experimentator.

Reporting for specific materials, systems and methods

We require information from authors about some types of materials, experimental systems and methods used in many studies. Here, indicate whether each material, system or method listed is relevant to your study. If you are not sure if a list item applies to your research, read the appropriate section before selecting a response.

Materials & experimental systems

n/a	Involved in the study	
☐	☒	Antibodies
☐	☒	Eukaryotic cell lines
☒	☐	Palaeontology and archaeology
☐	☒	Animals and other organisms
☒	☐	Clinical data
☒	☐	Dual use research of concern
☒	☐	Plants

Methods

n/a	Involved in the study	
☒	☐	ChIP-seq
☐	☒	Flow cytometry
☒	☐	MRI-based neuroimaging

Antibodies

Antibodies used

Immunoprecipitation (IP) and western blot (WB)
Human GPIbα antibody (clone SZ2) Beckman Coulter IM0409 WB: 1/1000; IP: 2 mg
Mouse GPIbα antibody (clone Xia.G5) Emfret M040-0 WB: 1/250; IP: 1 mg
Mouse GPIbα antibody (clone Xia.G7) Emfret M042-0 WB: 1/250; IP: 1 mg

Western blot
Alix antibody Abcam Ab117600 1/1000
Arp2c antibody Proteintech 15058-1-AP 1/1000
c-Mpl antibody RD systems AF1317 1/2000
Camk2γ antibody Proteintech 12666-2-AP 1/500
Cdc42 antibody Proteintech 10155-1-AP 1/1000
Cofilin antibody Cell signaling D3F9 1/1000
Dync1/2 antibody Proteintech 12219-1-AP 1/1000
Ehd3 antibody Proteintech 25320-1-AP 1/200
Filamin A antibody Abcam ab76289 1/2000
Fibrinogen antibody Proteintech 20645-1-AP 1/1000
Integrin αIIb antibody RD systems MAB4118 1/1000
Integrin β3 antibody BD Bioiscience 611141 1/5000
IQGAP1 antibody Santacruz SC-10792 1/500
Kindlin3 antibody Abcam 68040 1/1000
MAPRE2 antibody Proteintech 10364-1-AP 1/500
MICAL1 antibody Proteintech 14818-1-AP 1/1000
MKLP1 antibody Abcam Ab9259 1/1000
MTCO2 antibody Proteintech 55070-1-AP 1/1000
Myh9 antibody sigma M8064 1/1000
Myl9 antibody Proteintech 10906-1-AP 1/1000
Paxilin antibody Upstate 05-417 1/500
Piezo1 antibody Proteintech 28511-1-AP 1/1000
Plxb2 antibody Proteintech 10602-1-AP 1/1000
Rab35 antibody Proteintech 11329-2-AP 1/500
Rab8 antibody Proteintech 55296-1-AP 1/500
Rac1 antibody Santacruz sc-217 1/1000
RhoA antibody Proteintech 10749-1-AP 1/1000
Talin antibody Abcam 71333 1/1000
Vimentin antibody Proteintech 10366-1-AP 1/2000
VWF antibody Dako A0082 1/1000
WASP antibody Santacruz SC-13139 1/100
Zyxin antibody Proteintech 10330-1-AP 1/1000
α-actinin antibody Chemicon MAB1682 1/500
α-tubulin antibody Abcam Ab52866 1/2000
β-actin antibody RD systems MAB8929 1/20 000
β-tubulin antibody Sigma T4026 1/2000
β-tubulin antibody Abcam ab6046 1/500
γ-actin antibody Proteintech 11227-1-AP 1/2000
14-3-3 z Proteintech 14881-1-AP 1/1000
HRP goat anti-rabbit secondary antibody Jackson IR 111-035-003 1/20 000
HRP donkey anti-mouse secondary antibody Jackson IR 715-036-151 1/20 000

HRP goat anti-rat secondary antibody Jackson IR 112-035-175 1/5000

Flow cytometry

GPIbα antibody (clone Xia. G5) Emfret M040-1 1/5
GPIbβ antibody (clone Xia.C3) Emfret M050-1 1/5
αIIbβ3 antibody (clone Leo.F2) Emfret M025-2 1/5
α2 antibody (clone Sam.G4) Emfret M070-1 1/5
GPVI antibody (clone JAQ1) Emfret M011-2 1/5
JON/A antibody Emfret M023-1 1/5
P-selectin antibody (clone Wug.E9) Emfret M130-2 1/5
vWF antibody Dako A0082 1/142.5
Alexa Fluor-647 donkey anti-rabbit Invitrogen A31573 1/288

(Immuno)-fluorescence
FITC Rat anti-mouse GPIbα antibody (clone Xia.G7) Emfret M042-1 1/250
Rat anti-mouse Integrin β3 antibody (clone Luc.A5) Emfret M030-0 1/250
FITC Rat Anti-Mouse CD41 (clone MWReg30) BD Pharmingen 553848 1/50
Rabbit anti-laminin Sigma-Aldrich L9393 1/250
Texas-Red secondary antibody goat anti-rabbit Vector TI-100 1/500

in ivo
anti-GPIX antibody - in vivo labeling Emfret X488 5 mg/mice

Validation

Immunoprecipitation/Westernblot:
https://www.beckman.fr/reagents/coulter-flow-cytometry/antibodies-and-kits/single-color-antibodies/cd42b/im0409
https://yiqi-oss.oss-cn-hangzhou.aliyuncs.com/aliyun/technology/178406/295377.pdf
https://fnkprddata.blob.core.windows.net/domestic/data/datasheet/EMF/M042-0.pdf
https://www.abcam.com/en-us/products/primary-antibodies/alix-antibody-3a9-ab117600?srsltid=AfmBOopPwzeZFWXOGlLtzG5EDGjaulV_tXfHDqEoYnAiIYB-cZGi5xMe
https://www.ptglab.com/products/ARPC2-Antibody-15058-1-AP.htm?srsltid=AfmBOorUmmBstZXrNLV5L7CL2yuUd5jjFUBkt6zbA2zH5dC8o22eiMUh
https://www.rndsystems.com/products/mouse-thrombopoietin-r-tpo-r-antibody_af1317
https://www.ptglab.com/products/CAMK2G-Antibody-12666-2-AP.htm
https://www.ptglab.com/products/CDC42-Antibody-10155-1-AP.htm
https://www.cellsignal.com/products/primary-antibodies/cofilin-d3f9-xp-rabbit-mab/5175
https://www.ptglab.com/products/DYNC1I2-Antibody-12219-1-AP.htm
https://www.ptglab.com/products/EHD3-Antibody-25320-1-AP.htm
https://www.abcam.com/en-us/products/primary-antibodies/filamin-a-antibody-ep2405y-ab76289
https://www.ptglab.com/products/Fibrinogen-alpha-chain-Antibody-20645-1-AP.htm
https://www.rndsystems.com/products/mouse-integrin-alpha2b-cd41-antibody-386627_mab4118
https://www.bdbiosciences.com/en-us/products/reagents/microscopy-imaging-reagents/immunofluorescence-reagents/purified-mouse-anti-human-cd61.611140?tab=product_details
https://www.scbt.com/fr/p/iqgap1-antibody-h-109
https://doc.abcam.com/datasheets/inactive/ab68040/en-us/urp2-kindlin-3-antibody-ab68040.pdf
https://www.ptglab.com/products/MAPRE2-Antibody-10364-1-AP.htm
https://www.ptglab.com/products/MICAL1-Antibody-14818-1-AP.htm
https://doc.abcam.com/datasheets/inactive/ab9259/en-us/mklp1-antibody-ab9259.pdf
https://www.ptglab.com/products/COX2-Antibody-55070-1-AP.htm
https://www.sigmaaldrich.com/FR/fr/product/sigma/m8064
https://www.ptglab.com/products/MYL2-Antibody-10906-1-AP.htm
https://www.merckmillipore.com/FR/en/product/Anti-Paxillin-Antibody-clone-5H11,MM_NF-05-417
https://www.ptglab.com/products/PIEZO1-Antibody-28511-1-AP.htm
https://www.ptglab.com/products/PLXNB2-Antibody-10602-1-AP.htm
https://www.ptglab.com/products/RAB35-Antibody-11329-2-AP.htm
https://www.ptglab.com/products/RAB8A-Antibody-55296-1-AP.htm
https://datasheets.scbt.com/sc-217.pdf
https://www.ptglab.com/products/RHOA-Antibody-10749-1-AP.htm
https://www.abcam.com/en-us/products/primary-antibodies/talin-1-antibody-ab71333
https://www.ptglab.com/products/VIM-Antibody-10366-1-AP.htm
https://www.agilent.com/store/productDetail.jsp?catalogId=A008202-2
https://www.scbt.com/fr/p/wasp-antibody-b-9
https://www.ptglab.com/products/ZYX-Antibody-10330-1-AP.htm
https://www.sigmaaldrich.com/FR/fr/product/mm/mab1682
https://www.abcam.com/en-us/products/primary-antibodies/talin-1-antibody-ab71333
https://www.rndsystems.com/products/human-mouse-rat-beta-actin-antibody-937215_mab8929
https://www.sigmaaldrich.com/FR/fr/product/sigma/t4026
https://www.abcam.com/en-us/products/primary-antibodies/beta-tubulin-antibody-loading-control-ab6046
https://www.ptglab.com/products/ACTG1-Antibody-11227-1-AP.htm
https://www.ptglab.com/products/YWHAZ-Antibody-14881-1-AP.htm

Flow cytometry/IF
https://www.emfret.com/fileadmin/user_upload/Datasheets/M040-1_XiaG5.pdf

www.emfret.com/fileadmin/user_upload/Datasheets/M050-1_XiaC3.pdf
www.emfret.com/fileadmin/user_upload/Datasheets/M025-2_LeoF2.pdf
www.emfret.com/fileadmin/user_upload/Datasheets/M011-1_JAQ1.pdf
www.emfret.com/fileadmin/user_upload/Datasheets/M070-1_SamG4.pdf
www.emfret.com/fileadmin/user_upload/Datasheets/M023-2_JON_A.pdf
www.emfret.com/fileadmin/user_upload/Datasheets/M130-2_WugE9.pdf
www.emfret.com/fileadmin/user_upload/Datasheets/M042-1_XiaG7.pdfchrome-extension://efaidnbmnnnibpcajpcglclefindmkaj/
www.emfret.com/fileadmin/user_upload/Datasheets/M030-0_LucA5.pdf
www.bdbiosciences.com/content/dam/bdb/products/global/reagents/flow-cytometry-reagents/research-reagents/single-color-antibodies-ruo/553xxx/5538xx/553848_base/pdf/553848.pdf
www.sigmaaldrich.com/FR/fr/product/sigma/l9393

in vivo:
www.emfret.com/fileadmin/user_upload/Datasheets/X488.pdf

Eukaryotic cell lines

Policy information about cell lines and Sex and Gender in Research

Cell line source(s)	DAMI (MEGAKARYOBLAST, HUMAN) CRL-9792 ™, ATCC, https://www.atcc.org/products/crl-9792
Authentication	From commercial sources
Mycoplasma contamination	All cell lines were tested for mycoplasma contamination periodically, including immediatly upon receipt via MycoAlert Mycoplasma Testing Kit(Lonza). Results were always negative for mycoplasma contamination.
Commonly misidentified lines (See ICLAC register)	No commonly misidentified cell lines were in this study.

Animals and other research organisms

Policy information about studies involving animals; ARRIVE guidelines recommended for reporting animal research, and Sex and Gender in Research

Laboratory animals	mouse Mical1fl/fl mice were kindly provided by R. Jeroen Pasterkamp (Utrecht, Netherlands), and were crossed with mice carrying the Cre recombinase under the control of the platelet factor 4 (PF4) promoter (Tiedt, R., Schomber, T., Hao-Shen, H. & Skoda, R. C. Pf4-Cre transgenic mice allow the generation of lineage-restricted gene knockouts for studying megakaryocyte and platelet function in vivo. Blood 109, 1503–1506 (2007)
Wild animals	No wild animals
Reporting on sex	Both male and female mice were analyzed (4-16-week-old).
Field-collected samples	For mice studies, housing and experiments were done as recommended by French regulations and the experimental guidelines of the European Community. Animals were provided with food and water ad libitum; bedding was enriched with wood pieces for gnawing and kraft paper for nesting. The temperature and humidity in the housing rooms were strictly controlled and maintained within the following ranges: 21±3°C (18–24°C) for temperature, and 50–60%±10% (45–65%) for relative humidity. A 12h/12h light/dark cycle was maintained throughout the study. All procedures were performed with constant attention to minimizing discomfort and pain (monitoring of endpoints, use of anesthesia). Animals were observed daily to ensure their well-being. Furthermore, all personnel involved in the project were technically qualified and received continuous training in animal experimentation practices. To minimize stress, mice were removed from the housing area only immediately before experimentation. Animals were never housed alone (a maximum of five per cage, with aggressive animals being excluded). Anesthesia, experimental procedures, and euthanasia were carried out in rooms completely separate from the housing area. Both male and female mice were analyzed (4-16-week-old). In all experiments, mice were anesthetized either with a mixture of ketamine and xylazine (100 mg/kg and 10 mg/kg, respectively) injected intraperitoneally or with inhaled isoflurane gas. This project was approved by the local ethical committee CEEA26 and the French government under the number APAFIS#25086-2020032312267714.
Ethics oversight	This project was approved by the local ethical committee CEEA26 and the French government under the number APAFIS#25086-2020032312267714.

Note that full information on the approval of the study protocol must also be provided in the manuscript.

Plants

Seed stocks

Report on the source of all seed stocks or other plant material used. If applicable, state the seed stock centre and catalogue number. If plant specimens were collected from the field, describe the collection location, date and sampling procedures.

Novel plant genotypes

Describe the methods by which all novel plant genotypes were produced. This includes those generated by transgenic approaches, gene editing, chemical/radiation-based mutagenesis and hybridization. For transgenic lines, describe the transformation method, the number of independent lines analyzed and the generation upon which experiments were performed. For gene-edited lines, describe the editor used, the endogenous sequence targeted for editing, the targeting guide RNA sequence (if applicable) and how the editor was applied.

Authentication

Describe any authentication procedures for each seed stock used or novel genotype generated. Describe any experiments used to assess the effect of a mutation and, where applicable, how potential secondary effects (e.g. second site T-DNA insertions, mosiacism, off-target gene editing) were examined.

Flow Cytometry

Plots

Confirm that:

☒ The axis labels state the marker and fluorochrome used (e.g. CD4-FITC).

☒ The axis scales are clearly visible. Include numbers along axes only for bottom left plot of group (a 'group' is an analysis of identical markers).

☒ All plots are contour plots with outliers or pseudocolor plots.

☒ A numerical value for number of cells or percentage (with statistics) is provided.

Methodology

Sample preparation

DAMI cells were grown in RPMI1640 medium with Glutamax supplemented with 10% fetal bovine serum and 1% Penicillin/Streptomycin in 5% CO2 condition at 37°C. For MICAL1 silencing, DAMI cells were transduced with lentiviral particles expressing MICAL1 shRNA as previously described28 or Luciferase shRNA as control (in pTRIP IZIE-GFP vector) with a multiplicity of infection of 15. Transduction efficiency was evaluated by flow cytometry with green fluorescent protein (GFP) as reporter expression.
Mouse platelet preparation
Blood was collected by cardiac puncture of anaesthetized mice with 80 uM D-phenylalanyl-L-prolyl-L-arginine chloromethyl ketone (PPACK) and 10% (vol/vol) ACD-C buffer (124 mM sodium citrate, 130 mM citric acid, 110 mM dextrose, pH 6.5). Washed platelets were isolated by centrifugation as previously described and resuspended in Tyrode's buffer and then 2 mM Ca2+ were added before platelet activation.

Flow cytometry analysis
Surface glycoproteins expression and surface b-galactose were measured in diluted whole blood (1/20 with Tyrode's buffer) using appropriate fluorophore-conjugated antibodies for GPIba, GPIbb, GPIX, integrin aIIb3, integrin a2, GPVI or Ricinus communis Agglutinin (RCA-I) lectin . Platelet activation was evaluated in washed platelets (3 x 108/mL) with a range of several agonists for 10 min without stirring at room temperature (RT). Activation level was evaluated by using JON/A (active conformation of mouse integrin aIIb3), and by measuring the P-selectin exposure. Platelets were incubated with antibodies or RCA-1 lectin for 20 min at RT and then diluted with PBS. Acquisition was performed with 10 000 events in the platelet gate.
DAMI cells, 10^6 cells were centrifuged and resuspended in 50µL de buffer A (0.5% BSA, 2 mM EDTA in PBS, pH 7.2) and appropriate fluorophore-conjugated antibodies. Cells were incubated 10 min at 4°C before being washed with buffer A and centrifuged, and the pellet was resuspended with 600 µL of buffer A and then acquired with the flow cytometer with 20 000 events in the gate of living cells.

Instrument

Samples were analyzed with an Accuri C6 cytometer (BD Biosciences).

Software

C6 plus analysis software (BD Biosciences)

Cell population abundance

platelets <96%
DAMI 100% with <50% of living cells

Gating strategy

FSC-H/SSC-H was used to discerned cells from background. Isotype control were used to discern negative to positive labelling

☒ Tick this box to confirm that a figure exemplifying the gating strategy is provided in the Supplementary Information.
